# Long noncoding RNA AURKAPS1 potentiates malignant hepatocellular carcinoma progression by regulating miR-142, miR-155 and miR-182

**DOI:** 10.1038/s41598-019-56036-3

**Published:** 2019-12-23

**Authors:** Jianhua Li, Wenzhi Guo, Wenping Xue, Pengfei Xu, Zhen Deng, Danhua Zhang, Shouhua Zheng, Xinguang Qiu

**Affiliations:** Department of General Surgery, The First Affiliated Hospital of Zhengzhou University, No.1 Jianshe East Road, Zhengzhou, Henan 450052 P.R. China

**Keywords:** Cell migration, Cancer metabolism

## Abstract

The mitotic serine/threonine kinase aurora kinase-A (AURKA) has been identified as carcinogenic in hepatocellular carcinoma (HCC). AURKAPS1, a long non-coding RNA (lncRNA), is the pseudo-gene of AURKA, which play important roles in the cancer. Its underlying functions and mechanisms in liver cancer progression remain largely unknown. The mRNA expression of AURKAPS1 in HCC tumor tissues was significantly higher, which is associated with tumor size and TNM stage. The high expression of AURKAPS1 promotes cell movement, migration and invasion. AURKAPS1 can increases the protein expression of RAC1, promotes the activation of ERK, and enhance the formation of membrane ruffles by binding with miR-182, miR-155 and miR-142 competively. Thus, AURKAPS1 could be a useful marker, and the combination of AURKAPS1/miRNAs (miR-142, miR-155 and miR-182) may be a new theoretical basis for the treatment of HCC.

## Introduction

Hepatocellular carcinoma (HCC) is a lethal cancer with increasing frequency in the world in the recently decades. More than 700,000 new cases happened, and approximately 600,000 people die of liver cancer every year^1–3^. Despite medical technology development and progress that have led to great achievements in the comprehensive treatment of liver cancer, liver cancer recurrence and metastasis result in prognoses that are not optimistic^4–6^. Therefore, it is significant to study the underlying mechanisms of HCC progression to provide new information for improving HCC treatments.

Long noncoding RNAs (lncRNAs) are a kind of the most important noncoding RNAs which has more than 200 nucleotides in length and don’t have the ability to code proteins^7–9^. LncRNAs were reported to paly emportant roles in lots of diseases spatially and temporally, especially in cancers, which indicating specific functions for lncRNAs^10–14^. Additionally, lncRNAs can modulate the functions of target microRNAs (miRNAs) by acting as a competitive endogenous RNA (ceRNA)^15^.

AURKAPS1 is a long noncoding RNA, also the pseudogene of AURKA, which is located in the intron region of RAB3 GTPase activating non-catalytic protein subunit 2 (RAB3GAP2) on chromosome 1. The expression and functions of AURKAPS1 in tumours have not been reported. LncRNAs can function as a ceRNA or as a molecular sponge to modulate the expression and biological functions of miRNA, for example, via regulating the post-transcriptional proceed. Thus, an important crosstalk may exist between lncRNA and miRNA. However, whether AURKAPS1 affects tumourigenesis by regulating miRNAs remains unclear.

In this project, we found the expression of AURKAPS1 was significantly higher in HCC tissues and cell lines, also AURKAPS1 potentiates the invasion and metastasis of HCC cells by regulating miRNAs, so it may become a potential target for the treatment of HCC in the future.

## Results

**Figure 1:**
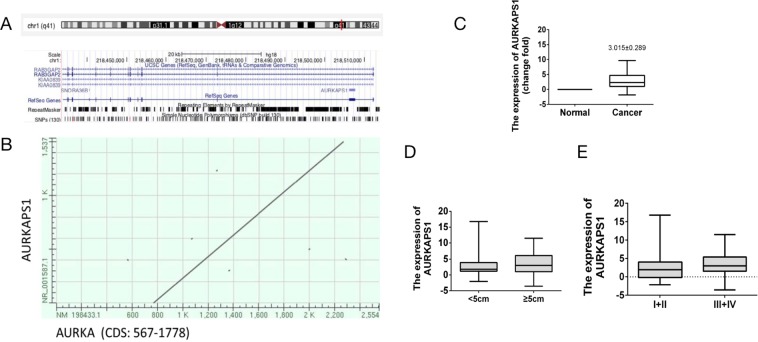
(**A**) Gene structure and localization of AURKAPS1. (**B**) Sequence comparison of AURKA and AURKAPS1. (**C**) Relative expression of AURKAPS1 in 124 pairs of HCC tissues (p = 0.0012)). (**D**) Relative expression of AURKAPS1 in different tumour sizes(p = 0.0241). (**E**) Relative expression of AURKAPS1 in different TNM stages(p = 0.0227). AURKAPS1 expression was examined by qRT-PCR. P < 0.05 was considered to indicate a statistically significant difference. The means ± SD are shown. Statistical analysis was conducted using Student’s t-test.

AURKAPS1 is the pseudogene of AURKA and is located in the intron region of RAB3GAP2 on chromosome 1 (Fig. 1A). Nevertheless, the expression and function of AURKAPS1 in tumours have not been reported. Through sequence comparison, we found that compared with the AURKA gene, the AURKAPS1 gene lacks a 359–560 coding region sequence, has a 25 bp difference in its 3′ extremity, and has some nucleotide mutations and losses (Fig. 1B). Furthermore, AURKAPS1 expression in 124 cases liver cancer tissues was detected by quantitative real-time PCR (qRT-PCR), and the results showed that AURKAPS1 expression was significantly higher in HCC tissues than in adjacent normal liver tissues (Fig. 1C). In addition, the expression level of AURKAPS1 was positively correlated with tumour size and TNM stage (Fig. 1D,E), but not with sex, age, history of hepatitis, or lymph node metastasis (Table 1), suggesting that AURKAPS1 may be associated with tumour invasion and metastasis.

**Table 1:** Relationship between AURKAPS1 expression and the clinicopathologic features of liver cancer patients.

Clinicopathologic features	No	Foldchange (Mean ± SD)	P value
**Sex**			
male	93	2.99 ± 3.18	0.626
female	31	3.33 ± 3.54	
**Age**			
≥50 years old	81	2.66 ± 2.92	0.052
<50 years old	43	3.86 ± 3.75	
**History of hepatitis**			
no	64	3.52 ± 3.45	0.120
yes	60	2.61 ± 3.01	
**Tumour size**			
<5 cm	59	2.47 ± 2.85	0.006*
≥5 cm	65	3.63 ± 3.53	
**Lymph node metastasis**			
no	99	2.90 ± 3.15	0.240
yes	25	3.76 ± 3.67	
**TNM stage**			
I, II	49	2.35 ± 3.50	0.046*
III, IV	75	3.55 ± 3.03	

*P < 0.05 compared with the control group. The means ± SD are shown. Statistical analysis was conducted using one-way analysis of variance (ANOVA).*P < 0.05 compared with the control group. The means ± SD are shown. Statistical analysis was conducted using one-way analysis of variance (ANOVA).

**Figure 2:**
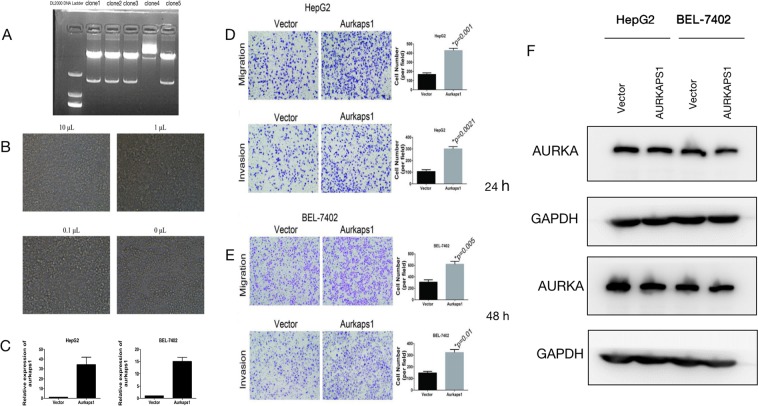
(**A**) Enzyme digestion identification of lentivirus plasmids. (**B**) Detection of cell activity after infection with lentivirus at different doses. (**C**) The expression of AURKAPS1 in stably transformed cells was detected byRT- PCR. (**D**) Effect of AURKAPS1 overexpression on HepG2 cell migration and invasion. (**E**) Effect of AURKAPS1 overexpression on BEL-7402 cell migration and invasion. (**F**) Effects of AURKAPS1 transfection with lentivirus on AURKA. The data are presented as the mean ± SD. *P < 0.05. Scale bars, 20 µm.

We consctruct AURKAPS1 Lentivirus vector using Ubi-MCS-SV40-EGFP-IRES-puromycin by Genechem Co. Adopt psPAX, pMD2.G entivirus packaging system to pack entivirus, then construct the AURKAPS1 overexpression stable cell lines, and identify them by RT-PCR (Fig. 2A–C). We evaluated cancer cell migration and invasion through Transwell assays. The migration and invasion of HepG2 and BEL-7402 cells were also significantly higher in the AURKAPS1-overexpression group than in the control group (Fig. 2D–G). These results indicated that AURKAPS1 may act as an oncogene to promote the migration and invasion of HCC cells.

When AURKAPS1 was overpressed in HepG2 and BEL-7402 cell lines, in order to prove whether the expression of AURKA was affected, we tested the expression of AURKA in both cell lines at 24 and 48h after transfection by western blot, and found there were no change for AURKA protein after the overexpression of AURKAPS1 (Fig. 2F).

**Figure 3:**
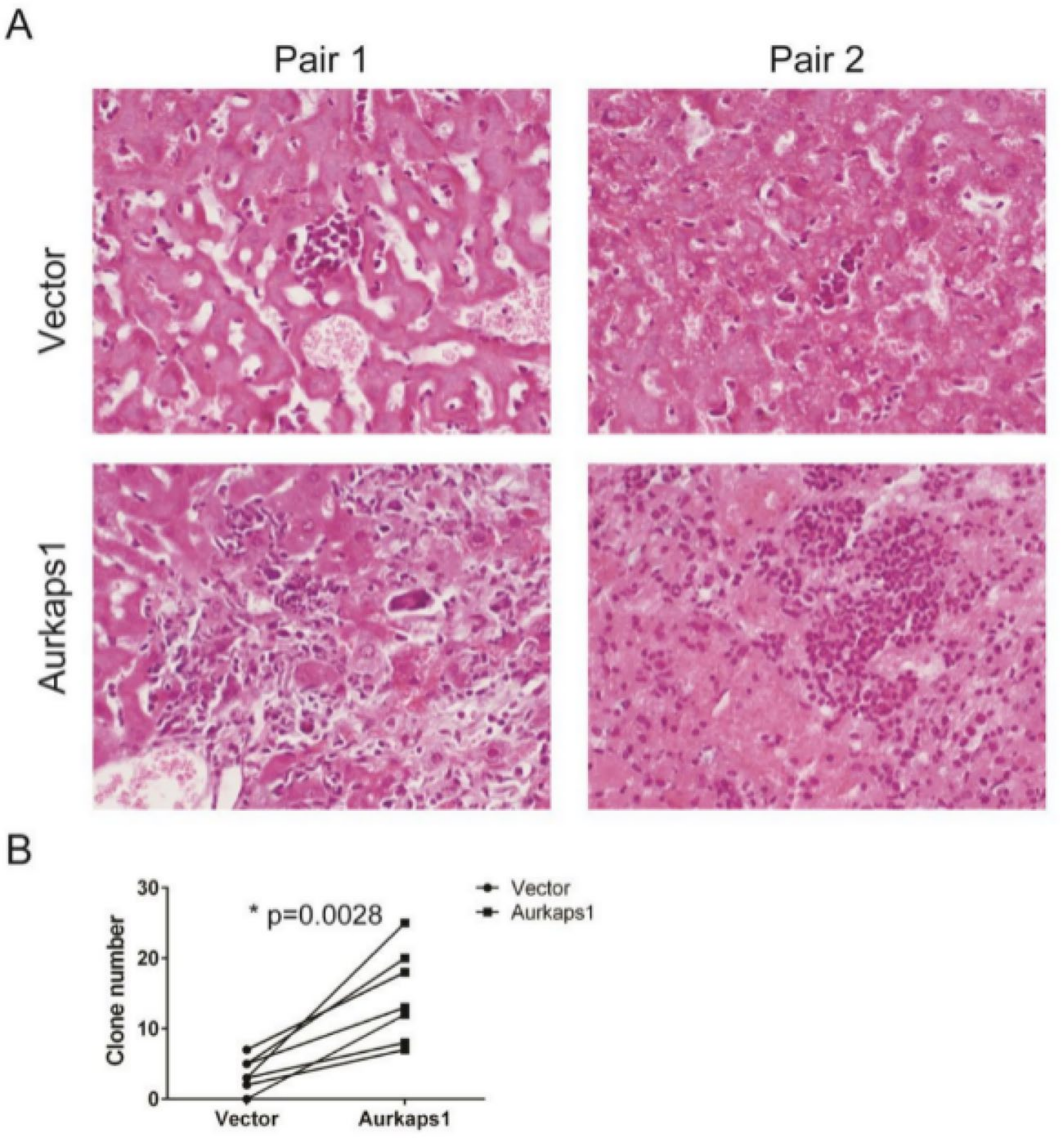
AURKAPS1 overexpression promotes liver carcinoma cell metastasis *in vivo*. (**A**) HE staining of liver tissue. (**B**) Number of tumour clones in liver tissue. *P < 0.05. Statistical analysis was conducted using Student’s t-test.

Invasion and metastasis are the cruces of malignant tumour recurrence. We simulated liver cancer metastasis by injecting HCC cells into the tail veins of mice. This metastasis tumour burden assay in the liver showed that the number and diameter of tumour nodules were significantly fewer and smaller in the control group than in the AURKAPS1 overexpression group (Fig. 3). Taken together, these *in vivo* results demonstrated that AURKAPS1 plays a crucial role in liver cancer cell migration and invasion.

**Figure 4:**
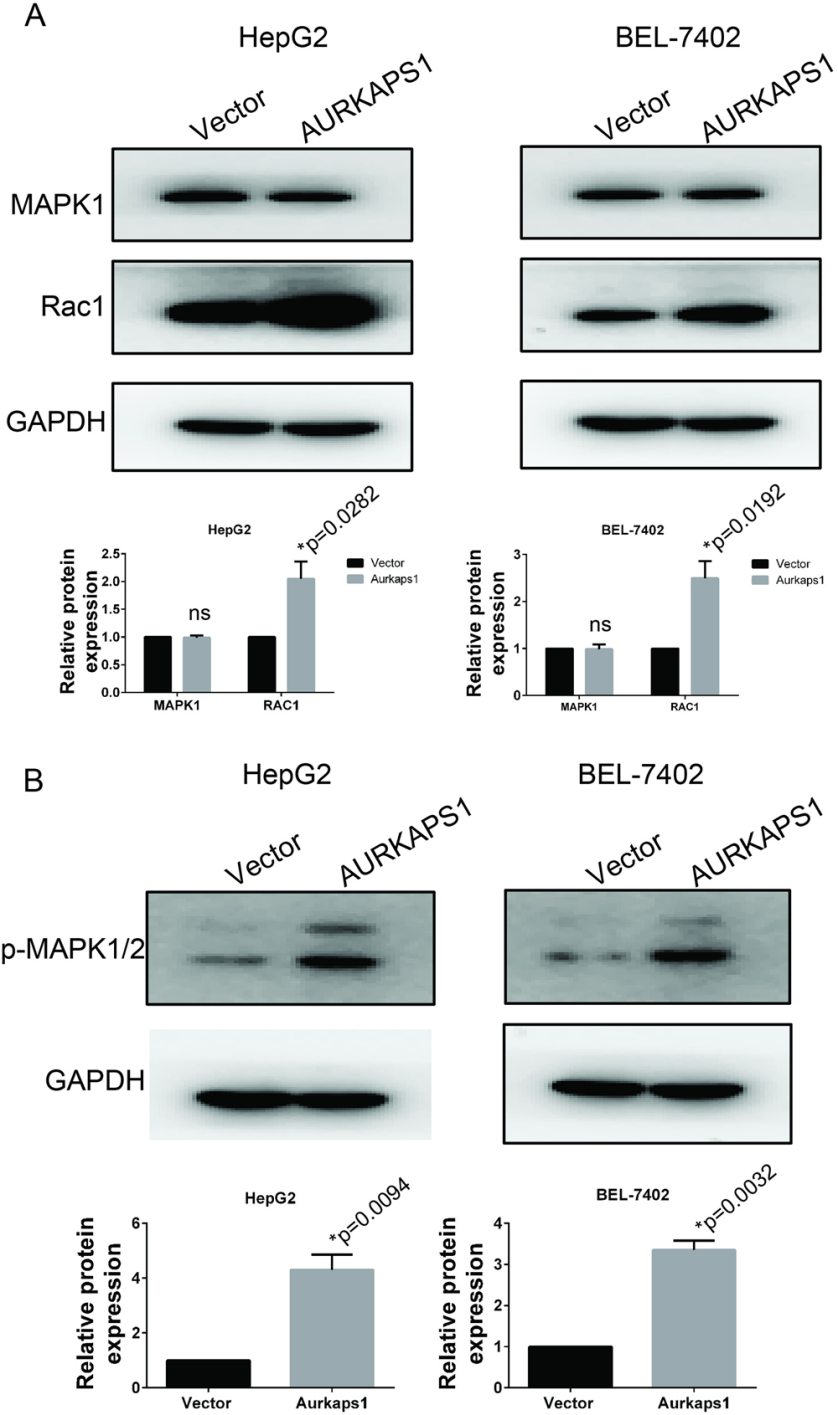
Western blotting was employed to determine RAC1, MAPK1 and p-MAPK1/2 expression. (**A**) Effect of AURKAPS1 overexpression on the RAC1 and MAPK1 protein. (**B**) Effect of AURKAPS1 overexpression on phosphorylated MAPK1/2 (p-MAPK1/2). The data are presented as the mean ± SD. *P < 0.05.

Through DIANA software analysis, we found that AURKAPS1 had ten miRNA binding sites (miR-134, miR-302, miR-636, miR-192, miR-182, miR-640, miR-1912, miR-767, miR-452 and miR-218). Functional bioinformatics prediction was then performed on the potential target genes of these miRNAs. By functional cross analysis, we identified six hub target genes (NM _000601 HGF, NM _000875 IGF1R, NM _002745 MAPK1, NM _006908 RAC1, NM _005359 Smad4, and NM _003392 Wnt5A). These results also suggest that AURKAPS1 might be involved in regulating the expression of these six genes as a ceRNA. To further verify the above hypothesis, we selected RAC1 and MAPK1, which are associated with tumour invasion and metastasis, as study subjects. Western blot analysis showed that AURKAPS1 overexpression could upregulate the RAC1 protein, but not MAPK1 (Fig. 4A). Because RAC1 can activate the MAPK1 pathway, we used phosphorylated MAPK1 antibodies to detect its active form. The results suggest that AURKAPS1 overexpression promoted the activation of MAPK1 (Fig. 4B).

**Figure 5:**
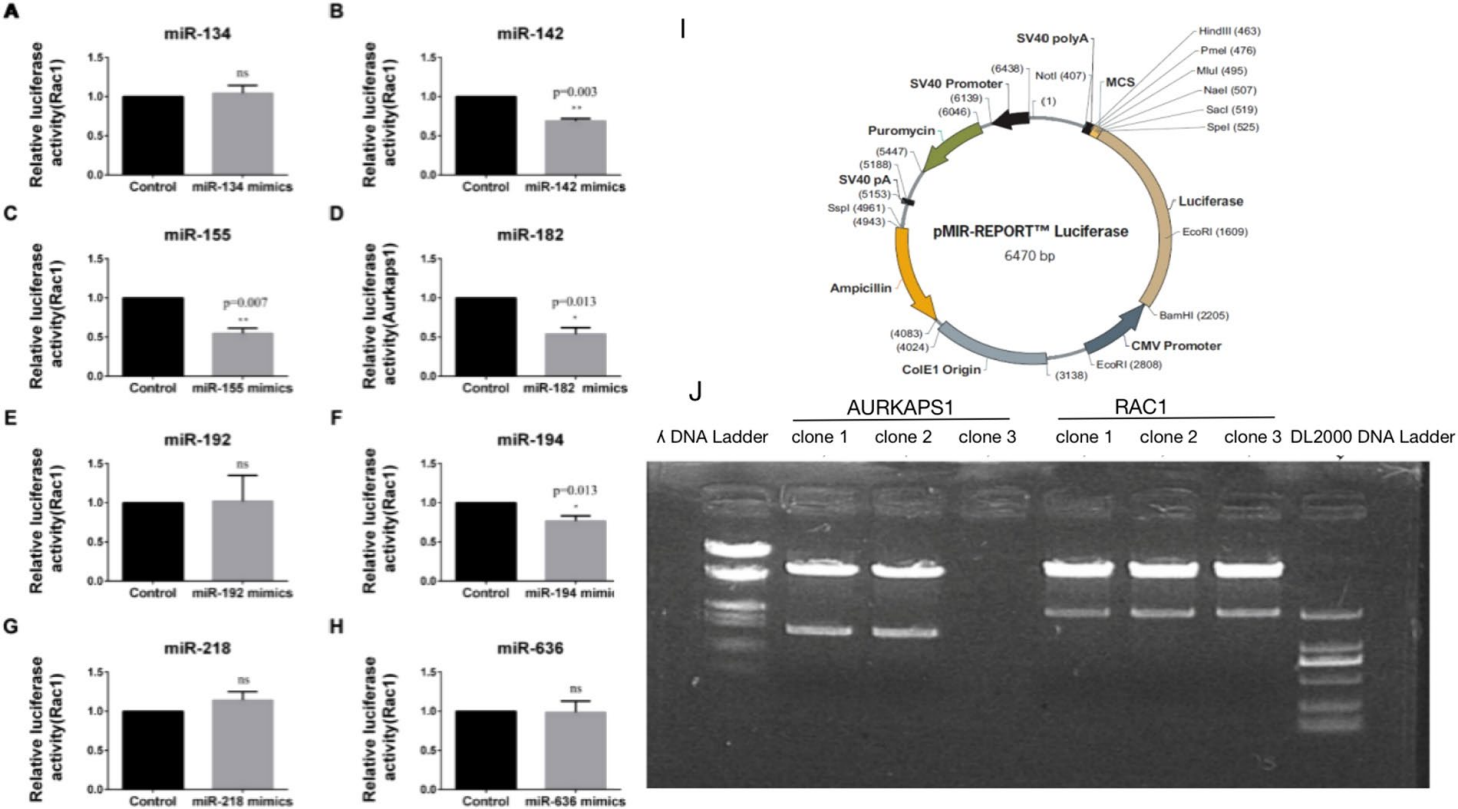
Relative RAC1 3′UTR luciferase activity in HepG2 cells treated with miRNAs. (**A**–**H**) Relative luciferase activity of the RAC1 3′UTR in HepG2 cells lines transfected with miR-134/miR-142/miR-155/miR-182/miR-192/miR-194/miR-218/miR-636. (**I**) Reporter gene vector map and construction. (**J**) Identification of AURKAPS1 and RAC1 3′utr reporter recombinant plasmids. Luciferase activity of the RAC13′UTR was examined by luciferase reporter gene assay. *P < 0.05, **P < 0.01. The means ± SD are shown. Statistical analysis was conducted using Student’s t-test.

We constructed RAC1 3′UTR and AURKAPS1 reporter gene plasmids to confirm which miRNA regulates RAC1 and AURKAPS1. In this study, five potential miRNA binding sites of AURKAPS1 (miR-134, miR-182, miR-192, miR-218 and miR-636) predicted by DIANA software and three underlying binding sites of the RAC1 3′UTR (miR-142, miR-155 and miR-194) predicted by TargetScan were selected for analysis (the above three binding sites were also present in AURKAPS1 as analysed by RNA22). The luciferase reporter gene assay indicated that miR-142, miR-155, miR-182 and miR-194 could suppress the reporter gene activity of RAC1 when the RAC1 3′UTR was cotransfected with the selected potential miRNAs (Fig. 5B–D,F), while miR-134, miR-192, miR-218 and miR-636 had no significant differences (Fig. 5A,E,G,H).

**Figure 6:**
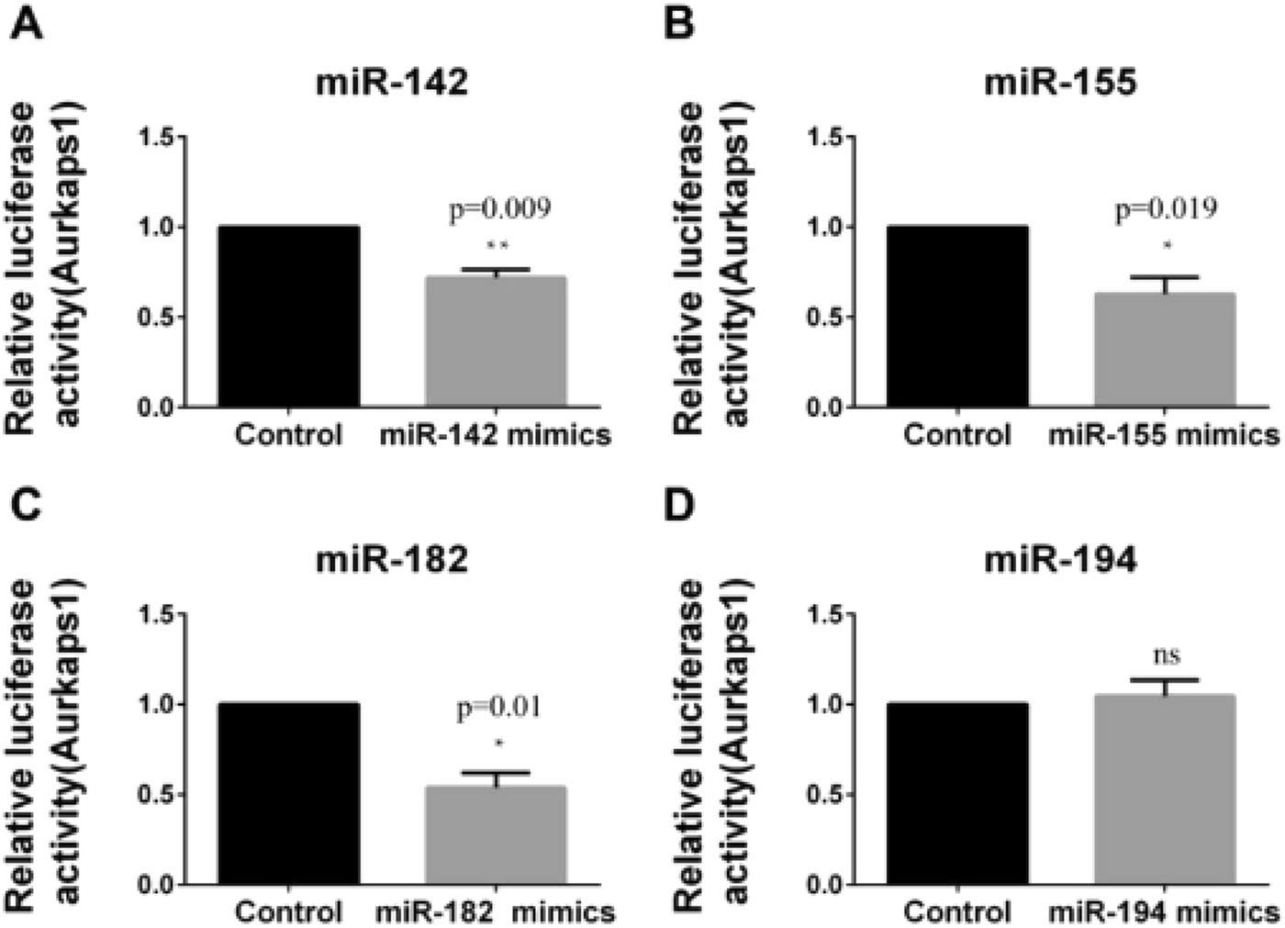
Relative AURKAPS1 luciferase activity in HepG2 cells treated with miRNAs. (**A**–**D**) Relative luciferase activity of AURKAPS1 in HepG2 cells lines transfected with miR-142./miR-155/miR-182./miR-194. The luciferase activity of AURKAPS1 was examined by luciferase reporter gene assay. *P < 0.05, **P < 0.01. The means ± SD are shown. Statistical analysis was conducted using Student’s t-test.

Subsequently, the luciferase reporter gene assay was used to analyse whether the 4 screened miRNAs that regulated RAC1 could simultaneously bind to AURKAPS1. We found that miR-142, miR-155 and miR-182 could also significantly inhibit the luciferase activity of AURKAPS1 (Fig. 6A–C), while miR-194 had no significant effect on the luciferase activity of AURKAPS1 (Fig. 6D).

**Figure 7:**
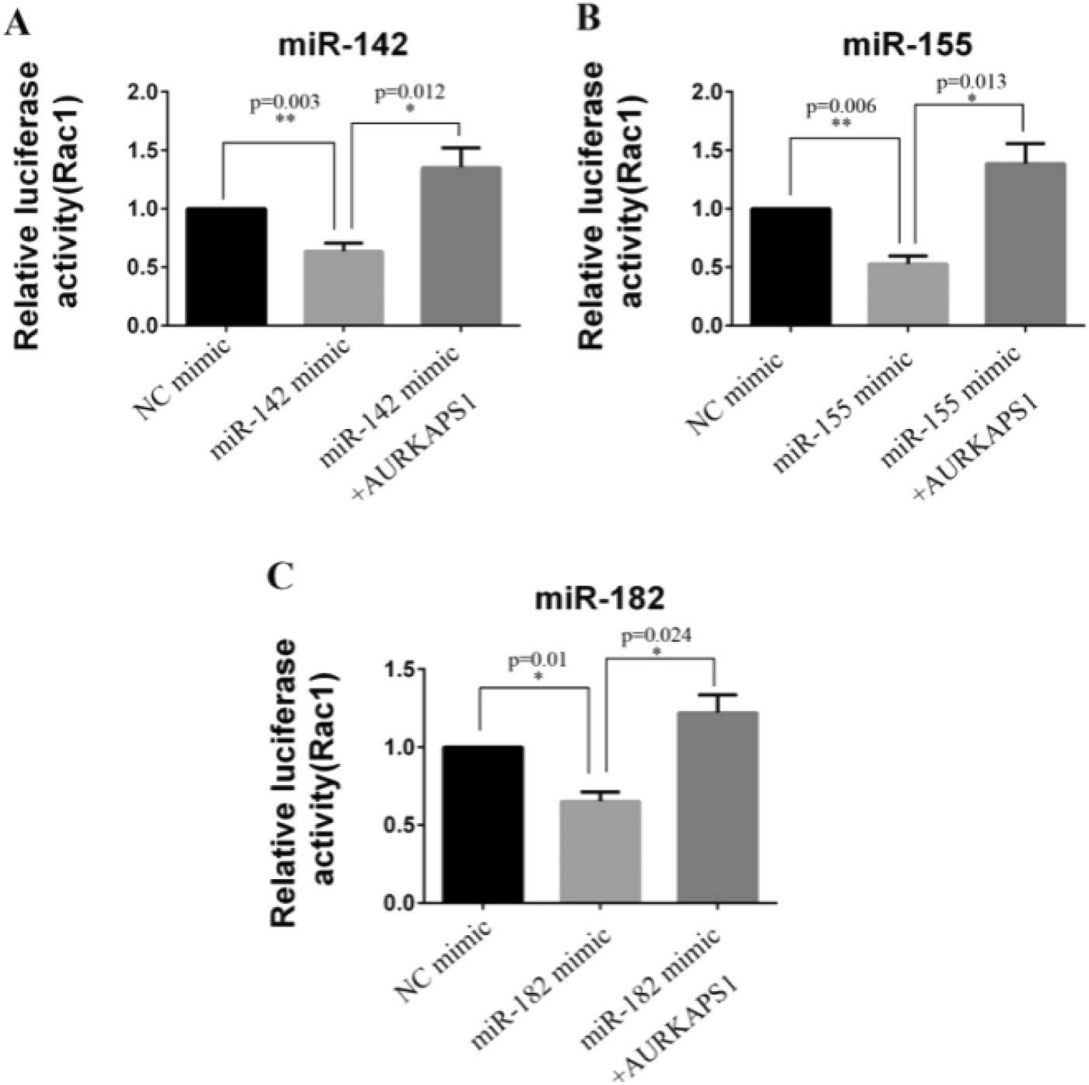
Relative RAC13′UTR luciferase activity in HepG2 cells treated with miRNAs/miRNAs+ AURKAPS1. (**A**) Relative luciferase activity of the RAC1 3′UTR in HepG2 cells lines transfected with miR-142/miR-142+ AURKAPS1. (**B**) Relative luciferase activity of the RAC1 3′UTR in HepG2 cells lines transfected with miR-155/miR-155+ AURKAPS1. (**C**) Relative luciferase activity of the RAC1 3′UTR in HepG2 cells lines transfected with miR-182/miR-182+ AURKAPS1. The RAC1 3′UTR luciferase activity was examined by luciferase reporter gene assay. *P < 0.05, **P < 0.01. The means ± SD are shown. Statistical analysis was conducted using Student’s t-test.

As lncRNA can regulate the function of miRNA as a ceRNA, we conducted further reporter gene experiments to verify whether AURKAPS1 can be used as a competitive binding molecule to control the regulation of RAC1 by miRNA. The results revealed that the luciferase activity of RAC1 decreased when miR-142 (Fig. 7A), miR-155 (Fig. 7B) and miR-182 (Fig. 7C) were overexpressed, and this suppression was significantly weakened when AURKAPS1 was overexpressed at the same time. These data confirmed that AURPAPS1 could suppress the targeting effect of miR-142, miR-152 and miR-182 on RAC1 via binding to miRNAs, thus improving the luciferase activity of RAC1.

**Figure 8:**
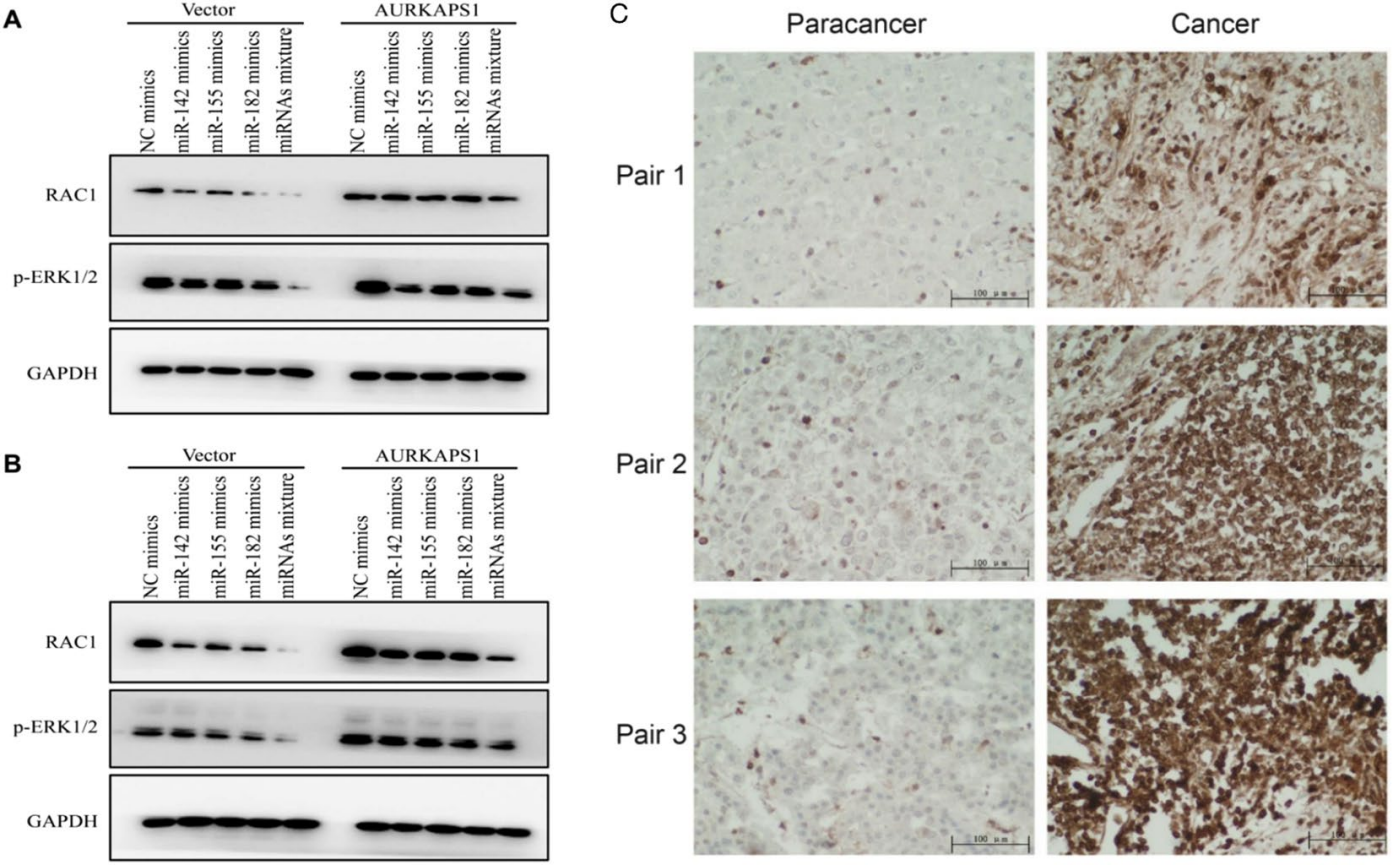
(**A**) Effect of AURKAPS1 overexpression in HepG2 cells on RAC1 and phosphorylated ERK1/2 downregulation by miR-142, miR-155 and miR-182. (**B**) Effect of AURKAPS1 overexpression in BEL-7402 cells on RAC1 and phosphorylated ERK1/2 downregulation by miR-142, miR-155 and miR-182. (**C**) Immunohistochemistry was employed to determine RAC1 expression in liver cancer tissues. Scale bars, 100 µm.

Western blot experiments were performed to further validate the competitive effect of AURKAPS1. RAC1 protein expression was significantly decreased when miR-142, miR-155 and miR-182 were overexpressed in the control cells. Moreover, the decrease in RAC1 levels was greater when these three miRNAs were cotransfected than when single miRNAs were transfected. The targeted inhibitory effect of these miRNAs on RAC1 was partially restored in AURKAPS1-overexpressing cells. This result also confirmed that AURKAPS1 could competitively inhibit the effects of miRNA downregulation (miR-142, miR-155 and miR-182) on RAC1 and promote ERK activation (Fig. 8A,B).

AURKAPS1 expression is significantly increased in HCC tissues and can promote RAC1 protein expression by competitively sponge miR-142, miR-155 and miR-182. We detected RAC1 protein expression in 100 pairs of liver cancer tissues through immunohistochemistry. The results indicated that RAC1 expression was also significantly higher in liver cancer tissues than in adjacent normal tissues (Fig. 8C).

## Discussion

Recently, lots of studies focused on the functions and the regulatory mechanisms of lncRNAs^16,17^, people have revealed that lncRNAs took part in various human cancers progression^18–20^. However, the functions and mechanisms of AURKAPS1 in HCC have not been reported, and remains unknown.

In the present project, we found that AURKAPS1 acted as an oncogenic biomarker in HCC. AURKAPS1 can target to miR-142, miR-155 and miR-182, and there was a negative correlation between AURKAPS1 and miRNAs. AURKAPS1 served as a ceRNA to potentiate cancer progress partially due to its ability to suppress the expression of miR-142, miR-155 and miR-182. Hence, our results was significance to improve the theoretical basis for lncRNAs in cancer therapy.

Emerging evidence has proved that many kinds of lncRNAs are abnormally expressed in different cancers^21^. Because lncRNAs take part in the occurrence and progression of malignant tumor, lncRNAs could be used as diagnostic or prognostic markers and potential therapeutic targets. A previous study indicated that HULC was upregulated in hepatoma and promoted cell proliferation, migration and invasion^10^. We observed similar findings in this study, AURKAPS1 overexpression accelerated the malignant progression of hepatocellular carcinoma cells. Also, we explored RAC1 expression and found that AURKAPS1 overexpression significantly improved RAC1 levels in liver cancer cells. RAC1 activation and mutation are closely related to the onset of melanoma and non-small cell lung cancer^22^. RAC1 overexpression can activate the PAK or ERK pathway to enhance tumour growth and aggressiveness^23^. In addition, RAC1 was found to be strongly linked to F-actin assembly and implicated in epithelial-to-mesenchymal transition (EMT)^24^. However, whether RAC1 is also involved in the AURKAPS1-induced enhancement of liver cancer progression needs to be further studied.

To date, AURKAPS1 and its underlying regulatory mechanisms have remained largely unknown. The ceRNA hypothesis may explain the mechanism of the complex biological function of lncRNA in the pathogenesis of human cancer partly^25–30^, also provides a basic theory to predict the potential functions of novel lncRNAs. Interestingly, we found the underlying molecular mechanism of how AURKAPS1 participates in liver cancer progression, it functions as a ‘molecular sponge’ to regulate miRNAs. Many studies have demonstrated lncRNAs play a vital role in a variety of cell processes by acting as ceRNAs to regulate miRNAs^8,15^, such as lncRNA HULC and MEG3^31–33^, which have been studied in several cancer research. In this study, we evaluated the effect of AURKAPS1 on liver cancer cells and discovered that AURKAPS1 took part in the development of pseudogene-miRNA-mRNA interaction networks and acted as an endogenous miRNA sponge, binding to miRNA and regulating its function. AURKAPS1 was confirmed to be a direct target gene for miR-142, miR-155 and miR-182, and RAC1 may be a potential regulatory target of AURKAPS1. Studies have indicated that miR-142/155/182 inhibits many cancers, such as lung cancer^34,35^, pancreatic cancer^36–38^, liver cancer^39,40^.

In conclusion, we found that the expression of AURKAPS1 and miRNAs (miR-142, miR-155 and miR-182) showed a significantly negative correlation in HCC cell lines. These three miRNAs remarkably downregulated AURKAPS1, while AURKAPS1 overexpression reversed the RAC1 reduction induced by miR-142, miR-155 and miR-182. Our results indicate that high AURKAPS1 expression promotes the tumourigenesis and progression of liver cancer through regulating miRNA expression. Thus, AURKAPS1 could be a useful marker, and the combination of AURKAPS1/miRNAs (miR-142, miR-155 and miR-182) may be a promising choice for the therapyof human liver cancer.

## Materials and Methods

### Sample preparation

All samples for the project were collected from patients who had undergone surgery and were diagnosed with liver cancer based on a pathological evaluation at the First Affiliated Hospital of Zhengzhou University. No other treatment related to HCC had been proceeded in the patients before their surgical treatment. All the specimens were immediately snap-frozen and preserved in liquid nitrogen then transfered to the −80 °C refrigerator until used in this study. All patients signed the informed consent form. This research was conducted by the Declaration of Helsinki. Human investigations were approved by the Institutional Review Board of the First Affiliated Hospital of Zhengzhou University. All animal procedures and experiments methods were carried out according to the Guide for the Care and Use of Laboratory Animals and were approved by the Institutional Animal Care and Use committee of the First Affiliated Hospital of Zhengzhou University.

#### Cell lines and culture

Human liver cancer cell lines HepG2 and BEL-7402 were purchased from the Shanghai Cell Bank of the Chinese Academy of Science (Shanghai, China). These cells were maintained in high-glucose Dulbecco’s modified Eagle’s medium (DMEM-H) (HyClone Co, USA) containing 10% FBS in a humidified atmosphere of 5% CO_2_ at 37 °C.

#### Cell transfection

The AURKAPS1 overexpression plasmid and the respective vector (negative control) were synthesized by GenePharma Co. (Shanghai, China). Cells were transiently transfected using Lipofectamine 2000 transfection reagent (Life Technologies Corp., Shanghai, China) according to the manufacturer’s protocol. The transfection efficiency was tested by qRT-PCR analysis.

#### RNA extraction and qRT-PCR

Total RNA was extracted from frozen liver samples and cells with TRIzol reagent (Invitrogen, Carlsbad, CA, USA). cDNA was synthesised from RNA using the 50 μl systerm with an RNA reverse transcription kit, diluted 1:10, and stored in −20 °C refrigerator (Takara Co., Dalian, China). Using One-Step SYBR Prime Script RT-PCR kit for qRT-PCR, and the endogenous control was GAPDH. The relative expression level (change fold) was calculated using the 2^−ΔΔCt^ method. The formula was reffered to the ABI Prism 7300 sequence detection system protocol.

#### Cell migration and invasion experiments

In order to test cell migration, place 8-mm pore size culture inserts (Transwell; Corning Costar, USA) into 24-well culture plates, upper and the lower chambers were seperated. For the lower chamber, add DMEM containing 10% FBS. Place serum-free medium containing 5 × 10^4^ cells in the upper chamber to examine migration and invasion. Then incubate at 37 °C for 48 h, the cells on the upper membrane surface were scraped off. The cells on the lower side of the membrane were fixed and then stained with 0.4% trypan blue dye. The number of cells that had migrated through the pores was quantified by counting 10 independent visual fields (magnification x20) under the microscope and then subjected to statistical analyses. Each experiment was performed at least 3 times.

#### Construction of luciferase reporter plasmid

Select pMIR-REPORTTM Luciferase as the reporter gene vector, and the vector information is discribed in Fig. 5I,J. The reporter vector was digested according to the following reaction system (Lentivirus vector plasmid 1 μg, SpeI incision enzyme 1 μL, MluI incision enzyme 1 μL, Rapid enzyme digestion buffer 3 μL, add Sterilization of water to 30 μL) to obtain the desired enzyme digestion vector.

### PCR amplification of the target sequence

RNA sequences of RAC1 and AURKAPS1 were downloaded from the NCBI nucleic acid database, and then Primer Premier software was used to design reporter primers of RAC1 and AURKAPS1, respectively. Meanwhile, restriction enzyme sites were added according to sequence characteristics, and PCR primers were used to amplify RAC1’s 3′UTR (non-coding region of 3′ segment) and AURKAPS1, respectively.

RAC1 3′UTR upstream primer:(include SpeI enzyme loci):AGACTAGTATGTCTCAGCCCCTCGTTCTT; RAC1 3′UTR downstream primer(include Mul I enzyme loci):TTACGCGT TATGATTCAAGGATTTATTAAGTCATACAT; AURKAPS1upstream primer(include SpeI enzyme loci): AGACTAGT TCTCCAGTCACAAGCCAGTTCAG; AURKAPS1 downstream primer(include Mul I enzyme loci): TTACGCGTACTATATCTCTAGCAGCTGTCCACAGT.

Put the reaction solution into the PCR amplification instrument and perform PCR amplification according to the procedure. After amplification, separate PCR reaction products by 1% agarose gel electrophoresis, and cut the target fragment under UV light, and use agarose gel recovery kit to recover the target fragment. The recovered products were digested by enzymes. At last, connect the target fragment and the carrier according to the reaction system (Recombinant plasmid 1 μl, SpeI incision enzyme 0.5 μl, MluI incision enzyme 0.5 μl, Rapid enzyme digestion buffer 1 μl, add Sterilization of water to 10 μL). Using 1% agarose gel electrophoresis to separate, and acquire the image under ultraviolet lamp.

#### Dual-luciferase repoerter gene assay

A biological information website was used to predict the promoter sequence of miRNA genes that may bind to AURKAPS1. After PCR amplification, the miRNA gene promoter sequence was inserted into a luciferase reporter gene vector to construct a luciferase reporter plasmid. Next, the miRNA promoter luciferase reporter plasmid and AURKAPS1 plasmid were cotransfected into HepG2 cells. Utilizing the Dual-Luciferase Reporter Assay System to exzamine the luciferase activity after 48–72 h to observe the changes in the fluorescence value.

### Western blot

After extacting the total protein from the cell, prepare 10% SDS-page gel, reheat the diluted sample at 55 °C for 5min, then add 15 μl sample to each well, and 7 μl maker in the first well. After adding the running buffer, Electrophoresis was performed at 90 V constant pressure for 30 minutes and 110 V for 70 minutes. PVDF film of 8.5 cm × 5.5 cm size was cut and soaked with methanol for 1 min. Then transfer the film at a constant pressure of 100 V under the condition of ice bath for 1.5 h. Rinsed with methanol again with TBST 5 min × 4 times, and sealed with TBST containing 5% milk at room temperature for 1 h. Add the properly diluted primary antibody according to the antibody instructions and incubate at 4 °C overnight. Second day, appropriately diluted peroxidase-labeled secondary antibody was added, and incubated at room temperature for 1 h. The incubated protein membrane was washed with TBST buffer for 5 min × 4 times. Put the film into the tray of the ECL gel imager, drop the appropriate amount of ECL substrate onto the film, click the appropriate exposure time and collect the image.

#### Tumour xenografts

Six weeks old female nude BALB/7 mice were purchased from Beijing Weitong Lihua Laboratory Animal Co, Ltd. (Beijing, China). The mice were given free access to sterile food and water during the experimental period. All animal experiments using nude mice were performed strictly in accordance with a protocol approved by the Beijing Research Center of Laboratory Animals. The mice were divided to twogroups (n = 7 in each group). The first group is tumor invasion experiment group, the mice were injected 1 × 10^6^/100 μL l HepG2 (over expressed AURKAPS1) cells with a 1 ml syringe through the tail vein; the second group is the control groupcontrol group cells (1 × 10^6^/100 μl PBS) were injected 0.8 weeks after the transplantation, sacrifice all the mice to harvest the liver to detect the tumor metastasis.

### Immunohistochemistry

A continuous section of the wax (3–4 μm thickness) mass with tumour tissue was selected for immunohistochemical staining. A sodium citrate buffer solution (0.01 M, pH 6.0) was heated in a pressure cooker and kept warm for antigen repair. After blocking with 5% normal goat serum and 0.2% Triton X-100 in PBS for 1 h at room temperature, the liver sections were incubated overnight at 4 °C with 1% normal goat serum and 0.2% Triton X-100 in PBS containing primary antibodies against RAC1 to visualize hepatoma cells (rabbit, 1:500; Abcam, USA). After washing, the sections were then incubated with the species-appropriate biotin-labelled secondary antibodies overnight at 4 °C or for 2 h at room temperature. The slides were then coated with glycerol and covered for microscopic analysis.

#### Statistical analysis

All the data are showed as the mean ± standard deviation (SD). All experimental results were statistically analysed with Student’s t-test or one-way analysis of variance (ANOVA). All statistical analyses were performed with Graphad prism 8.0 version. A value of P < 0.05 was considered to indicate a statistically significant difference.
